# Selecting a viral load threshold for routine monitoring in resource‐limited settings: optimizing individual health and population impact

**DOI:** 10.1002/jia2.25007

**Published:** 2017-11-24

**Authors:** Tanya M Ellman, Bereket Alemayehu, Elaine J Abrams, Stephen Arpadi, Andrea A Howard, Wafaa M El‐Sadr

**Affiliations:** ^1^ Mailman School of Public Health ICAP at Columbia University New York NY USA; ^2^ Division of Infectious Diseases Department of Medicine College of Physicians and Surgeons Columbia University New York NY USA; ^3^ Department of Pediatrics College of Physicians and Surgeons Columbia University New York NY USA; ^4^ Department of Epidemiology Mailman School of Public Health Columbia University New York NY USA

**Keywords:** viral load, HIV monitoring, resource limited setting, threshold, antiretroviral therapy, scale‐up

The routine use of HIV viral load tests for monitoring patients on antiretroviral therapy (ART) in resource‐limited settings has the potential to greatly benefit those with HIV infection as well as public health in general. Declines in viral load after ART initiation result in improved clinical outcomes 1, 2 and viral load testing has therefore been considered the gold standard for measuring ART response in high‐resource settings for many years 3. Reductions in viral load also reduce the risk of perinatal 4 and sexual transmission of HIV 5. However, availability of viral load testing has been limited in resource‐limited settings where less sensitive and specific clinical and immunologic measures have largely been used to determine response to therapy; as of 2013 it was estimated that less than 20% of ART patients in Africa receive routine viral load testing 6, 7. In the past few years, enormous efforts have been made to scale‐up availability of viral load testing for routine monitoring, however, some countries remain with little access, others are testing low proportions of patients on ART, and only a few are testing the majority of their ART patients 8, 9. Further, the impact of this recent viral load scale‐up on clinical and public health outcomes are yet to be determined.

While initial studies in resource‐limited settings found no mortality benefit when comparing viral load to CD4 count for monitoring 10, 11, the rationale for monitoring viral load in patients on ART is based on several factors that pertain to both individual patient management as well as optimizing population health. Viral load testing can find individuals who might benefit from additional adherence support and leads to earlier and more accurate identification of treatment failure than clinical and immunologic indicators 10, 12, 13. This allows for appropriate antiretroviral regimen selection and improved outcomes for individual patients, and provides a public health benefit through more appropriate allocation of second‐line medications. Prompt detection of treatment failure through viral load monitoring may also prevent emergence of drug resistance mutations, providing benefit to the individual in settings where antiretroviral drug options are limited, and also at the population level by limiting transmission of drug‐resistant virus 14.

The World Health Organization (WHO) guidelines recommend the use of viral load as the preferred method for monitoring treatment response over clinical and immunological approaches, and define virologic failure with a threshold of 1000 copies/ml 7, 15. The selection of this threshold has generated debate as to whether the desired goals of routine viral load monitoring, on individual and public health levels, will be achieved by using the 1000 copies/ml threshold and whether it will prove optimal across patient populations such as infants, children, adolescents, and pregnant and breastfeeding women.

In most resource‐rich contexts, the goal of ART is to achieve viral suppression, usually defined as below the limit of detection of the assay (e.g. <20, <25, <37, <40 copies/ml) 16. While studies show that transient episodes of viremia that subsequently return to below the limit of detection, often called “blips,” do not predict subsequent virologic failure 17, 18, persistent low‐level viremia carries some increase in risk of emergence of drug resistance and subsequent virologic failure 18, 19. Data show that persistent low‐level viremia between 50 and 999 copies/ml, especially at the higher end of that range, is associated with an increased risk of resistance mutations, particularly M184I/V and K103N 19, which may impact effectiveness of first‐line regimens most commonly used in resource‐limited settings. Persistent viremia below 1000 copies/ml has also been noted to be associated with an increased risk of virologic failure 18, including with viral load levels as low as 50 to 199 copies/ml that persist for at least six months 20.

At the same time, there is also evidence to support the selection of a viral load threshold of 1000 copies/ml, particularly in resource‐limited settings. Though the optimal value is not known, a viral load below 1000 copies/ml is associated with a low risk of disease progression 21 and with a decrease in HIV transmission. Available data demonstrate that sexual transmission is very unlikely with a viral load <1700 copies/ml and even less likely with a viral load <400 copies/ml 22, 23, 24, and mother‐to‐child transmission is around one percent in women on antiretroviral drugs with a viral load <1000 copies/ml 4. As a result, using a threshold of 1000 copies/ml appears to provide both individual and public health benefits while also simplifying the approach to routine viral load monitoring.

The choice of threshold for viral load among patients on ART in resource‐limited settings is also influenced by available technology for viral load measurement. Use of dried blood spots (DBS) for viral load testing is a promising approach which offers advantages related to ease of specimen collection and handling, and allows for specimen transport without a cold chain, however, its use also impacts the choice of viral load threshold. Adapting laboratory viral load assays to accommodate DBS specimens poses specific technical challenges, such as lower sensitivity due to the lower specimen volume, differences in efficiency of nucleic acid extraction, and presence of amplification inhibitors such as hemoglobin 25. As a result, lower limits of detection using DBS are much higher than those of plasma even for the same assay. In addition, amplification of cell‐associated DNA or RNA in DBS reduces the specificity of DBS viral load testing 25.

The specific issues related to DBS measurement led to an initial reluctance to use the 1000 copies/ml threshold for DBS. Thus, the WHO 2013 guidelines suggest considering a threshold of 3000 to 5000 copies/ml for such specimens 15. However, advances in technology such as the introduction of RNA‐specific extraction and amplification procedures to commercial kits have improved the specificity of DBS‐based viral load testing 26. Furthermore, a systematic review demonstrated acceptable performance characteristics for DBS compared to plasma for most technologies at the 1000 copies/ml threshold 7. Therefore, the recent WHO guidelines recommend the threshold of 1000 copies/ml for viral load testing through DBS on most laboratory‐based platforms 7, 26, when there are operational barriers to using plasma. This threshold was recommended for all viral load methodologies, whether plasma or DBS‐based, in order to simplify the training of diverse clinical providers and to enable the consistent and accurate implementation of viral load monitoring. However, there is an urgent need for additional data on the performance of DBS specimens using a viral load threshold of 1000 copies/ml in routine program settings, as well as data on the outcomes of patients with the use of this threshold for viral load monitoring.

Implementation of viral load testing for routine monitoring of ART response in resource‐limited settings has the potential to lead to improved individual patient outcomes as well as to decreased risk of HIV transmission, potentially changing the trajectory of the HIV epidemic. While treatment experts advocate for the use of the lowest possible viral load threshold as the goal of HIV treatment, those with interest in the public health impact of viral load monitoring support the use of the 1000 copies/ml threshold as a pragmatic choice. The latter is offered as a compromise between the ideal for the individual and the need for a focus on achieving the greater good.

Moving forward, it will be particularly important to carefully evaluate the effect of use of the 1000 copies/ml threshold in terms of individual and population impacts. Such data will be critically important in informing future recommendations and guidelines. Most importantly, while viral load scale‐up is an important priority, it is critical that it be coupled with ensuring access to such testing for all HIV‐positive patients on treatment and effective utilization of results, irrespective of the viral load threshold selected, in order to achieve the promise of HIV treatment.

## Competing interests

Authors have no competing interests to declare.
